# Supplementary material for: Topologically enhanced exciton transport
Source: Nat Commun. 2025 Dec 13;16:11448. doi: 10.1038/s41467-025-66276-9 (PMC12749365; doi:10.1038/s41467-025-66276-9)
Supplement: Supplementary file 1 — Supplementary Information [file 41467_2025_66276_MOESM1_ESM.pdf]

SUPPLEMENTAL MATERIAL
Topologically enhanced exciton transport

Joshua J. P. Thompson,[1] Wojciech J. Jankowski,[2] Robert-Jan Slager,[2] and Bartomeu Monserrat[1,2]

[1] *Department of Materials Science and Metallurgy, University of Cambridge, 27 Charles Babbage Road, Cambridge CB3 0FS, United Kingdom*

[2] *Theory of Condensed Matter Group, Cavendish Laboratory, University of Cambridge, J. J. Thomson Avenue, Cambridge CB3 0HE, United Kingdom*

(Dated: October 10, 2025)

I First principles calculations and numerical simulations

We perform density functional theory calculations using the Quantum Espresso package [1, 2] to study the polypentacene chain. We use kinetic energy cutoffs of 80 Ry and 500 Ry for the wavefunction and charge density, respectively, as well as 12 k-points to sample the Brillouin zone along the chain direction. Generalized gradient approximation norm-conserving pseudopotentials in the Perdew-Burke-Ernzerhof formulation are used as generated using the code ONCVPSP (Optimized Norm-Conserving Vanderbilt PSeudoPotential) [3]. These potentials can be found online via the Schlipf-Gygi norm-conserving pseudopotential library [4]. In order to prevent interactions between periodic images of the organic chain, we include a 34.3 Å spacing in the planar direction perpendicular to the organic polymer chain, as well as a vacuum spacing of 27.52 Å in the out-of-plane direction. Structural optimisation of the atomic coordinates was performed in order to reduce the forces below 0.0015 Ry/Å. The hopping parameters of the Su–Schrieffer–Heeger (SSH) tight-binding model [5, 6] were extracted from these calculations following Ref. [7]. We find $(t_1, t_2) = (0.87, 0.63)$ eV for polyanthracene, $(t_1, t_2) = (0.32, 0.53)$ eV for polypentacene and $(t_1, t_2) = (0.08, 0.41)$ eV for polyheptacene. To calculate the excitonic dispersions we follow the procedure outlined in our previous work [7]. For each pair of hopping constants (t_1, t_2), we solve the Wannier equation:

$$\left[\sum_{k'} (E^e_{k+Q/2} - E^h_{k-Q/2})\delta_{k,k'} - W_{k,-k',Q}\right] \psi^n_Q(k) = E_n(Q)\psi^n_Q(k). \tag{1}$$

obtaining the exciton energy $E_n(Q)$ and envelope function $\psi^n_Q(k)$ of the nth excitonic band. The electron/hole energies, $E^{e(h)}$, come from our SSH parameterisation, while the interaction matrix is:

$$W_{k,-k',Q} = V_{\mathrm{NR}}(k-k') \sum_{i,j\in\{A,B\}} \varphi^*_{i,k+Q/2}\varphi^*_{j,k'-Q/2}\varphi_{j,k-Q/2}\varphi_{i,k'+Q/2},$$

where $\varphi_{i,k}$ is the electronic wavefucntion on site i from the SSH model with momentum k. The Coulomb potential V_{NR} describing the screening in a one-dimensional system that captures the quasi 1D/2D nanoribbon [8],

$$V_{\mathrm{NR}}(Q) = \frac{e_0^2}{4\pi\epsilon_0} \frac{K_0(QL/2)}{\varepsilon_s + 8Q^2\alpha_{1\mathrm{D}}K_0(QL/2)}, \tag{2}$$

where L is the width of the ribbon, $\alpha_{1\mathrm{D}}$ is the screening parameter, ε_s is the background dielectric screening, and K_0 is a modified Bessel function of the second kind. We take L to be the lateral size of the nanoribbon and fix $\alpha_{1\mathrm{D}} = 0.05\,\mathrm{nm}^{-2}$ following previous work [8]. In this work, to simulate realistic experimental conditions we assume the polyacene nanoribbon is placed on an SiO_2 substrate with background dielectric $\epsilon_s = 2.45$ [9]. Using the resulting exciton wavefunctions and band structures we can numerically resolve all the equations shown in the main text. Further details on exciton-phonon and exction-electric field interactions are outlined in the methods section of the main text.

II Derivation of the free excitonic quantum Fokker-Planck dynamics

To describe free exciton diffusion, we assume that the static lattice of ions fully determines the exciton effective mass m^*_ν, where the mass is band-dependent. The corresponding effective continuum Hamiltonian driving free exciton propagation in one spatial dimension is:

$$\hat{H}_\nu = -\frac{\hbar^2}{2m^*_\nu}\partial_x^2. \tag{3}$$

Inserting the band-projected density $\hat{\rho}_\nu = \hat{P}_\nu \hat{\rho} \hat{P}_\nu$, where $\hat{P}_\nu = \sum_Q \left|u^{\text{exc}}_{\nu Q}\right\rangle \left\langle u^{\text{exc}}_{\nu Q}\right|$ is a projector onto the exciton band with index ν, we can write the associated Heisenberg equation:

$$\mathrm{i}\hbar \partial_t \, \hat{\rho}_\nu = [\hat{H}_\nu, \hat{\rho}_\nu]. \tag{4}$$

In an effective band-projected picture, the free excitonic density propagation driven by Heisenberg equation can be mapped to an exact partial differential diffusion equation governing the Fokker-Planck dynamics underpinned by Fick's law [10]. Upon employing the position representation, the density evolution reduces to the one-dimensional diffusion equation:

$$\partial_t \, \rho_\nu(x,t) = D_\nu \partial_x^2 \, \rho_\nu(x,t). \tag{5}$$

On mapping the Heisenberg equation to the diffusion equation [10], we associate the diffusivity of excitons with an inverse of their effective mass:

$$D_\nu = \frac{\hbar}{2m^*_\nu}. \tag{6}$$

With the diffusion equation or the band-projected exciton density $\rho_\nu(x,t)$, consistent with the Fick's law, we solve the differential equation to obtain a Gaussian solution for propagation:

$$\rho_\nu(x,t) = \frac{1}{\sqrt{2\pi[2D_\nu t + \sigma^2_{\text{Ini}}]}} \exp\left(-\frac{(x-x')^2}{2[2D_\nu t + \sigma^2_{\text{Ini}}]}\right). \tag{7}$$

Recognizing that the expected value of the density can be interpreted as a probability distribution, $\rho_\nu(x,t) \to P(x,x'|t,t')$, and shifting the initial time $t_{\text{ini}} = 0 \to t_{\text{ini}} = t'$, we explicitly retrieve a Fokker-Planck propagation for the excitons:

$$P(x,x'|t,t') = \frac{1}{\sqrt{2\pi[2D(t-t') + \sigma^2_{\text{Ini}}]}} \exp\left(-\frac{(x-x')^2}{2[2D(t-t') + \sigma^2_{\text{Ini}}]}\right), \tag{8}$$

with diffusivity being reflected by the quantum mechanics at the level of the effective mass.

III Derivation of the exciton diffusivity with quantum geometric contributions

In this section we derive the result for free exciton diffusivity quoted in the Methods of the main text, explicitly:

$$D_\nu = \frac{1}{2\hbar}\left\langle \frac{\partial^2 E_{\nu Q}}{\partial Q^2}\right\rangle + \frac{1}{\hbar}\sum_{\mu\neq\nu} \langle \Delta_{\mu\nu}(Q) g^{\mu\nu}_{xx}(Q)\rangle. \tag{9}$$

As derived in the previous section, the diffusivity of freely-propagating excitons is given by $D_\nu = \hbar/2m^*_\nu$. Following Ref. [11], we define the effective mass m^*_ν for a localized wavepacket in exciton band ν and in one dimension as:

$$\frac{1}{m^*_\nu} = \frac{1}{\hbar^2}\langle\langle \partial^2_Q H_{\text{exc}}\rangle\rangle_\nu \equiv \frac{1}{\hbar^2}\frac{\int_{\text{BZ}} \mathrm{d}Q \ \left\langle u^{\text{exc}}_{\nu Q}\right| \partial^2_Q H_{\text{exc}} \left|u^{\text{exc}}_{\nu Q}\right\rangle}{\int_{\text{BZ}} \mathrm{d}Q}, \tag{10}$$

where $H_{\text{exc}}= \frac{\hbar^2 Q^2}{2m^*_\nu} + V_{\text{eff}}(R)$ is an effective exciton Hamiltonian associated with the exciton band dispersion $E_\nu(Q)$ and the exciton Bloch states $\left|u^{\text{exc}}_{\nu Q}\right\rangle$, which experience an effective potential $V_{\text{eff}}(R)$. To connect the effective mass to the quantum geometry of excitons, we utilise the Hellmann-Feynman theorem:

$$\begin{aligned}\left\langle u^{\text{exc}}_{\nu Q}\right| \partial_Q H_{\text{exc}} \left|u^{\text{exc}}_{\nu Q}\right\rangle &= \partial_Q\Big(\left\langle u^{\text{exc}}_{\nu Q}\right| H_{\text{exc}} \left|u^{\text{exc}}_{\nu Q}\right\rangle\Big) + \left\langle u^{\text{exc}}_{\nu Q}\right| H_{\text{exc}} \left|\partial_Q u^{\text{exc}}_{\nu Q}\right\rangle + \left\langle \partial_Q u^{\text{exc}}_{\nu Q}\right| H_{\text{exc}} \left|u^{\text{exc}}_{\nu Q}\right\rangle \\ &= \partial_Q E_\nu(Q) + E_\nu(Q)\Big(\left\langle u^{\text{exc}}_{\nu Q}\middle|\partial_Q u^{\text{exc}}_{\nu Q}\right\rangle + \left\langle \partial_Q u^{\text{exc}}_{Q}\middle|u^{\text{exc}}_{\nu Q}\right\rangle\Big) \\ &= \partial_Q E_\nu(Q),\end{aligned} \tag{11}$$

where we use the product rule and the normalisation condition on the Bloch states $1 = \langle u^{\rm exc}_{\nu Q} | u^{\rm exc}_{\nu Q} \rangle \implies 0 = \partial_Q \Big(\langle u^{\rm exc}_{\nu Q} | u^{\rm exc}_{\nu Q} \rangle \Big) = \langle \partial_Q u^{\rm exc}_{\nu Q} | u^{\rm exc}_{\nu Q} \rangle + \langle u^{\rm exc}_{\nu Q} | \partial_Q u^{\rm exc}_{\nu Q} \rangle$. Also using the product rule for the second derivative, which enters the effective mass, we obtain:

$$\begin{aligned} \langle u^{\rm exc}_{\nu Q} | \, \partial^2_Q H_{\rm exc} \, | u^{\rm exc}_{\nu Q} \rangle &= \partial_Q \Big(\langle u^{\rm exc}_{\nu Q} | \, \partial_Q H_{\rm exc} \, | u^{\rm exc}_{\nu Q} \rangle \Big) - \langle u^{\rm exc}_{\nu Q} | \, \partial_Q H_{\rm exc} \, | \partial_Q u^{\rm exc}_{\nu Q} \rangle - \langle \partial_Q u^{\rm exc}_{\nu Q} | \, \partial_Q H_{\rm exc} \, | u^{\rm exc}_{\nu Q} \rangle \\ &= \partial^2_Q E_\nu(Q) - \sum_\mu \langle u^{\rm exc}_{\nu Q} | \, \partial_Q H_{\rm exc} \, | u^{\rm exc}_{\mu Q} \rangle \langle u^{\rm exc}_{\mu Q} | \partial_Q u^{\rm exc}_{\nu Q} \rangle - \sum_\mu \langle \partial_Q u^{\rm exc}_{\nu Q} | u^{\rm exc}_{\mu Q} \rangle \langle u^{\rm exc}_{\mu Q} | \, \partial_Q H_{\rm exc} \, | u^{\rm exc}_{\nu Q} \rangle , \end{aligned} \tag{12}$$

where we have inserted a resolution of the identity, $1 = \sum_\mu | u^{\rm exc}_{\mu Q} \rangle \langle u^{\rm exc}_{\mu Q} |$, in the second line. On differentiating the eigenvalue equation $H_{\rm exc} | u^{\rm exc}_{\nu Q} \rangle = E_\nu(Q) | u^{\rm exc}_{\nu Q} \rangle$, and taking an inner product with $\langle u^{\rm exc}_{\mu Q} |$, for $\mu \neq \nu$, we obtain:

$$\langle u^{\rm exc}_{\mu Q} | \, \partial_Q H_{\rm exc} \, | u^{\rm exc}_{\nu Q} \rangle = (E_\nu(Q) - E_\mu(Q)) \langle u^{\rm exc}_{\mu Q} | \partial_Q u^{\rm exc}_{\nu Q} \rangle = {\rm i} \Delta_{\mu\nu}(Q) A^{\rm exc}_{\mu\nu}(Q), \tag{13}$$

where $\Delta_{\mu\nu}(Q) = E_\mu(Q) - E_\nu(Q)$ is the energy difference between the pair of exciton bands μ and ν, and $A^{\rm exc}_{\mu\nu}(Q) \equiv {\rm i} \langle u^{\rm exc}_{\mu Q} | \partial_Q u^{\rm exc}_{\nu Q} \rangle$ is a non-Abelian excitonic Berry connection. Therefore, we can rewrite the previous condition as:

$$\begin{aligned} \langle u^{\rm exc}_{\nu Q} | \, \partial^2_Q H_{\rm exc} \, | u^{\rm exc}_{\nu Q} \rangle &= \partial^2_Q E_\nu(Q) + 2 \sum_{\mu \neq \nu} \Delta_{\mu\nu}(Q) \langle \partial_Q u^{\rm exc}_{\nu Q} | u^{\rm exc}_{\mu Q} \rangle \langle u^{\rm exc}_{\mu Q} | \partial_Q u^{\rm exc}_{\nu Q} \rangle - \partial_Q E_\nu(Q) \Big(\langle \partial_Q u^{\rm exc}_{Q} | u^{\rm exc}_{\nu Q} \rangle + \langle u^{\rm exc}_{\nu Q} | \partial_Q u^{\rm exc}_{\nu Q} \rangle \Big) \\ &= \partial^2_Q E_\nu(Q) + 2 \sum_{\mu \neq \nu} \Delta_{\mu\nu}(Q) g^{\mu\nu}_{xx}(Q), \end{aligned} \tag{14}$$

where the last term arises from the $\mu \neq \nu$ terms in the resolution of the identity, and we have also used the definition of the exciton quantum metric component $g^{\mu\nu}_{xx}(Q) = g^{\nu\mu}_{xx}(Q) \equiv \langle \partial_Q u^{\rm exc}_{\nu Q} | u^{\rm exc}_{\mu Q} \rangle \langle u^{\rm exc}_{\mu Q} | \partial_Q u^{\rm exc}_{\nu Q} \rangle$. Averaging over the exciton Brillouin zone (Q-space), we obtain:

$$\frac{1}{m^*_\nu} = \frac{1}{\hbar^2} \Big\langle \partial^2_Q E_\nu(Q) \Big\rangle + \frac{2}{\hbar^2} \sum_{\mu \neq \nu} \langle \Delta_{\mu\nu}(Q) g^{\mu\nu}_{xx}(Q) \rangle. \tag{15}$$

Inserting the relation between the effective mass and diffusivity $D_\nu = \hbar / 2 m^*_\nu$ into this expression, gives the final result:

$$D_\nu = \frac{1}{2\hbar} \left\langle \frac{\partial^2 E_{\nu Q}}{\partial Q^2} \right\rangle + \frac{1}{\hbar} \sum_{\mu \neq \nu} \langle \Delta_{\mu\nu}(Q) g^{\mu\nu}_{xx}(Q) \rangle. \tag{16}$$

We further comment on the scaling of the second term $\sum_{\mu \neq \nu} \langle \Delta_{\mu\nu}(Q) g^{\mu\nu}_{xx}(Q) \rangle$ with the band indices μ, ν. The metric reads:

$$g^{\mu\nu}_{xx}(Q) = \frac{\Big| \langle u^{\rm exc}_{\mu Q} | \, \partial_Q H_{\rm exc} \, | u^{\rm exc}_{\nu Q} \rangle \Big|^2}{|\Delta_{\mu\nu}(Q)|^2}, \tag{17}$$

and it immediately follows that $g^{\mu\nu}_{xx}(Q) \propto 1/|\Delta_{\mu\nu}(Q)|^2$. As a result, the combination of multiband metric and exciton gaps featuring in the diffusivity decays as $\Delta_{\mu\nu}(Q) g^{\mu\nu}_{xx}(Q) \propto 1/|\Delta_{\mu\nu}(Q)|$ with increasing $|\mu - \nu|$. Therefore, the interband contributions arising from the closest bands μ, ν are generally expected to dominate.

IV Comparison of Exciton Transport in Polypentacene, Polyanthracene and Polyheptacene

Previous works have shown that on increasing the number of rings in the polyacene polymer building block, a transition from trivial ($n = 3$) to topological ($n = 5$) electrons/excitons occurs [7, 12, 13]. In the main text we focus on the $n = 5$ polypentacene system as it is known to host topological electrons and excitons, however for completeness here we compare the exciton transport for polyanthracene ($n = 3$) and polyheptacene ($n = 7$), which host trivial/topological excitons, respectively. We extract t_1 and t_2 in the same way as in the polypentacene case, but for polyanthracene we have that $t_1 > t_2$. We plot the propagation of the exciton distribution in Fig. 1 for all three compounds.

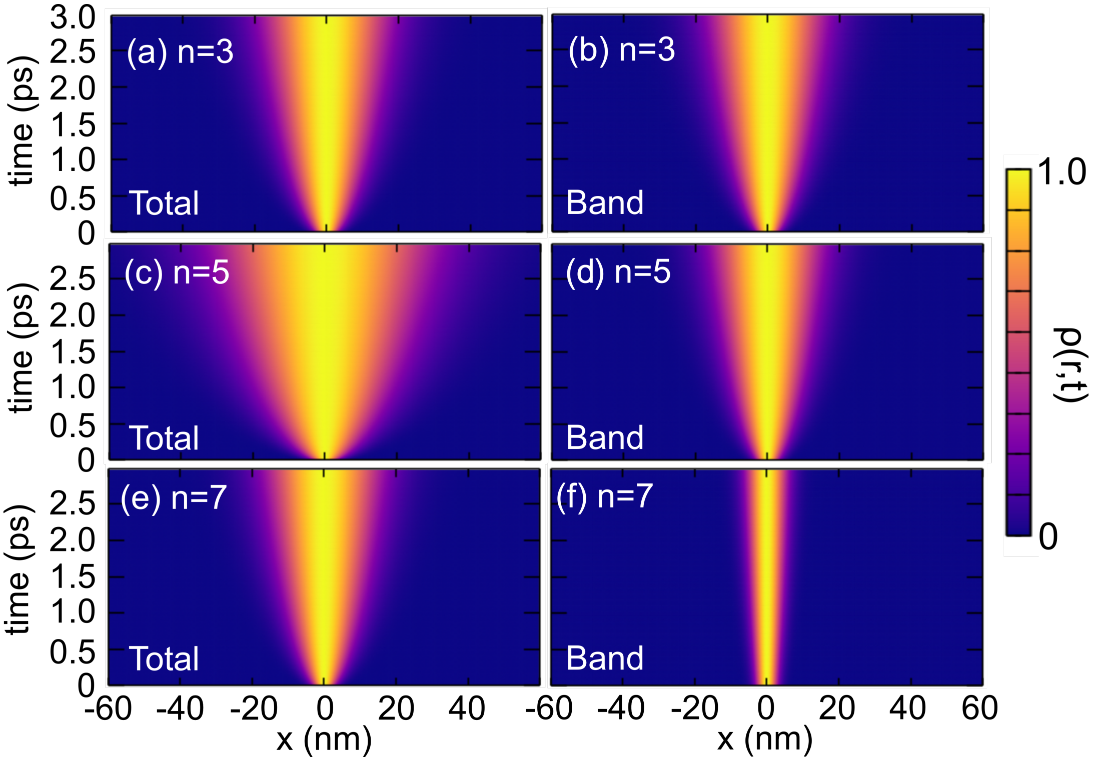

Supplementary FIG. 1. Comparison of the exciton diffusion in (a,b) polyanthracene ($n = 3$), (c,d) polypentacene ($n = 5$, Fig. 2, main text) and (e,f) polyheptacene ($n = 7$). Calculations were performed including the full geometric contribution in the left column (a,c,e) while only the band contribution was included in the right column (b,d,f).

Comparing first polyanthracene [Supplementary Fig. 1(a)] and polypentacene [Supplementary Fig. 1(c)] it is clear that the latter spreads significantly faster owing to the topological contribution to the exciton metric, as discussed in the main text. We find for polyheptacene [Supplementary Fig. 1(d)] a stark reduction in the exciton diffusion despite the $n = 7$ system hosting topological excitons [7, 12]. This can be attributed to the particularly flat bands of the electronics and excitonic dispersion in polyheptacene, leading to a reduction in both the band and geometric terms in exciton diffusion (Eq. 16). Without this geometry we calculate that the exciton diffusion would be a factor of 10 times smaller (0.022 cm^2 s^{-1} vs 0.19 cm^2 s^{-1}). Interestingly, the diffusion of polyheptacene is therefore similar to that of polyanythracene, despite the latter possessing much lower exciton mass/larger group velocity. To highlight the impact of the excitonic geometry we show in the right-hand column of Supplementary Fig. 1 the same diffusion neglecting geometric contributions. For polyanthracene [Supplementary Fig. 1(b)] the trivial excitons possess small metric so we see negligible change to the diffusion upon neglecting the metric. For the topological systems, polypentacene and polyheptacene, we find a clear drop in the exciton diffusion when geometry is neglected [Supplementary Fig. 1(d) and Supplementary Fig. 1(f), respectively]. The band in polypentacene (Supplementary Fig. 1d) is still quite dispersive leading to a sizeable diffusion in spite of the removal of the metric term. However, in the case of polyheptacene [Supplementary Fig. 1(f)], the band contribution is very small ($< 10\%$) leading to a pronounced difference in the diffusion. This system is closer to the flat-band limit discussed in the main text.

V Impact of band geometry on phonon-mediated diffusion

In this section we briefly outline the role that the geometric modification to the exciton group velocity has on the phonon-mediated diffusion. In the trivial case ($t_1 > t_2$) the geometric term leads to a modest increase in the exciton diffusion at low temperatures, while at larger temperatures the diffusion constant with and without the geometric term converge. This behaviour can be easily understood as follows: the geometric correction primarily increases the effective group velocity at and around $Q = 0$ where it is normally vanishing. Since the $Q = 0$ state is significantly more populated at low temperatures (assuming Boltzmann thermalisation), its importance becomes enhanced. Simultaneously, at low temperatures and low Q, the phonon-induced exciton dephasing Γ_Q is small leading

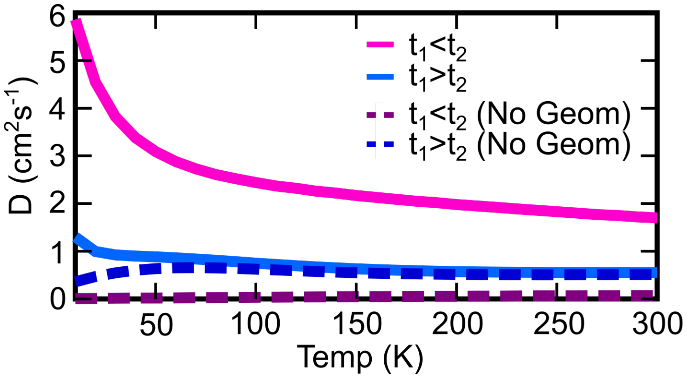

Supplementary FIG. 2. Exciton diffusion constant as a function of temperature with (solid) and without (dashed) quantum geometric modifications to the exciton group velocity. The solid lines correspond to those presented in the main text [c.f. Fig. 4(d)].

to a larger diffusion. At large temperatures, the relative population and hence importance of the $Q = 0$ state vanishes leading to convergence between the diffusion with and without geometry corrections.

In the case of the topological regime, $t_2 > t_1$, without geometric corrections to the velocity, the diffusion is significantly smaller ($\sim$ 5 times smaller) compared to the trivial regime, owing to the enhanced electron-phonon coupling. By including the geometry a massive increase in the exciton velocity, especially at low Q, leads to large enhancement of the diffusion *in spite* of the enhanced phonon scattering.

Supplementary References

[1] Giannozzi, P. *et al.* QUANTUM ESPRESSO: a modular and open-source software project for quantum simulations of materials. *Journal of Physics: Condensed Matter* **21**, 395502 (2009). URL http://dx.doi.org/10.1088/0953-8984/21/39/395502.

[2] Giannozzi, P. *et al.* Advanced capabilities for materials modelling with Quantum ESPRESSO. *Journal of Physics: Condensed Matter* **29**, 465901 (2017). URL http://dx.doi.org/10.1088/1361-648X/aa8f79.

[3] Hamann, D. Optimized norm-conserving Vanderbilt pseudopotentials. *Physical Review B* **88**, 085117 (2013). URL https://journals.aps.org/prb/abstract/10.1103/PhysRevB.95.239906.

[4] Schlipf, M. & Gygi, F. Optimization algorithm for the generation of ONCV pseudopotentials. *Computer Physics Communications* **196**, 36–44 (2015). URL https://www.sciencedirect.com/science/article/pii/S0010465515001897.

[5] Su, W. P., Schrieffer, J. R. & Heeger, A. J. Solitons in polyacetylene. *Phys. Rev. Lett.* **42**, 1698–1701 (1979). URL https://link.aps.org/doi/10.1103/PhysRevLett.42.1698.

[6] Su, W. P., Schrieffer, J. R. & Heeger, A. J. Soliton excitations in polyacetylene. *Phys. Rev. B* **22**, 2099–2111 (1980). URL https://link.aps.org/doi/10.1103/PhysRevB.22.2099.

[7] Jankowski, W. J., Thompson, J. J., Monserrat, B. & Slager, R.-J. Excitonic topology and quantum geometry in organic semiconductors. *Nature Communications* **16**, 4661 (2025). URL https://www.nature.com/articles/s41467-025-59257-5.

[8] Villegas, C. E. & Rocha, A. R. Screened hydrogen model of excitons in semiconducting nanoribbons. *Physical Review B* **109**, 165425 (2024). URL https://journals.aps.org/prb/abstract/10.1103/PhysRevB.109.165425.

[9] Thompson, J. J. *et al.* Anisotropic exciton diffusion in atomically-thin semiconductors. *2D Materials* **9**, 025008 (2022). URL https://iopscience.iop.org/article/10.1088/2053-1583/ac4d13/meta.

[10] Lee, M. H. Fick's law, Green-Kubo formula, and Heisenberg's equation of motion. *Phys. Rev. Lett.* **85**, 2422–2425 (2000). URL https://link.aps.org/doi/10.1103/PhysRevLett.85.2422.

[11] Onishi, Y. & Fu, L. Fundamental bound on topological gap. *Phys. Rev. X* **14**, 011052 (2024). URL https://link.aps.org/doi/10.1103/PhysRevX.14.011052.

[12] Cirera, B. *et al.* Tailoring topological order and π-conjugation to engineer quasi-metallic polymers. *Nature nanotechnology* **15**, 437–443 (2020). URL https://www.nature.com/articles/s41565-020-0668-7.

[13] Romanin, D., Calandra, M. & Chin, A. W. Excitonic switching across a $\mathbb{Z}_2$ topological phase transition: From Mott-Wannier to Frenkel excitons in organic materials. *Phys. Rev. B* **106**, 155122 (2022). URL https://link.aps.org/doi/10.1103/PhysRevB.106.155122.
